# Draft Genome Sequence of the Toluene-Degrading *Pseudomonas stutzeri* Strain ST-9

**DOI:** 10.1128/genomeA.00567-15

**Published:** 2015-06-04

**Authors:** Margarita Gomila, Antonio Busquets, Elena García-Valdés, Esti Michael, Rivka Cahan, Yeshayahu Nitzan, Jorge Lalucat

**Affiliations:** a Microbiologia, Departament de Biologia, Universitat de les Illes Balears, Palma de Mallorca, Spain; b Institut Mediterrani d’Estudis Avançats (IMEDEA, CSIC-UIB), Universitat de les Illes Balears, Palma de Mallorca, Spain; c Department of Chemical Engineering, Ariel University, Ariel, Israel; d The Mina and Everard Goodman Faculty of Life Sciences, Bar-Ilan University, Ramat Gan, Israel

## Abstract

Strain ST-9 was isolated from toluene-contaminated soil (Samaria, Israel). The draft genome has an estimated size of 4.8 Mb, exhibits an average G+C content of 60.37%, and is predicted to encode 4,183 proteins, including a gene cluster for aromatic hydrocarbon degradation. It is assigned to genomovar 3 of *Pseudomonas stutzeri*.

## GENOME ANNOUNCEMENT

Strain ST-9 was isolated from toluene-contaminated soil from the Barkan industrial zone in Samaria, Israel, after enrichment in a mineral medium with toluene as the only carbon and energy source. Phenotypic traits and a phylogenetic multilocus sequence analysis performed as described by Mulet and colleagues (1) demonstrated that *Pseudomonas stutzeri* was its closest neighbor.

Whole-genome shotgun sequencing on strain ST-9 was performed by using paired-end sequencing with a MiSeq sequencing system (Illumina). The Newbler Assembler version 2.7 software package (Roche) was used for the *de novo* genome assembly. The draft genome size is 4,643,775 bp and contains 116 contigs, with an average contig length of 40.03 kb, a median coverage depth of 75-fold, and an average G+C content of 60.37 mol%.

The genome prediction and annotation was performed using the NCBI Prokaryotic Genome Automatic Annotation Pipeline (http://www.ncbi.nlm.nih.gov/genome/annotation_prok/). Analysis and comparison of the functional annotation was done using the Kyoto Encyclopedia of Genes and Genomes (KEGG Automatic Annotation Server [KAAS]) (2). A total of 4,183 coding sequences, 52 tRNA sequences, and 1 rRNA sequence were identified in the chromosome. Genes coding for discriminating metabolic and physiological properties of the species were detected: the complete set of genes for the denitrification pathway, for starch metabolism, and for flagella synthesis. Of the 4,183 encoded proteins, 32 were annotated as transposases and 18 as integrases. Five hundred sixty-seven encoded proteins were annotated as hypothetical proteins without function prediction.

No plasmids were detected. Strain ST-9 was found to possess in contig 2, close to an integrase gene, a fragment of 19,158 bp organized as a putative operon with a regulatory gene and 21 genes related to an aromatic degradation pathway that includes the pathway to channel the metabolites to catechol and an aromatic ring cleavage enzyme with 75% similarity with the catechol 2,3 dioxygenase gene of the TOL plasmid pWW0. Genes coding for proteins involved in the resistance against arsenic, cobalt/zinc/cadmium, mercury, copper, and chromate were located in other 4 contigs, in the vicinity of transposase and integrase genes.

Whole-genome sequences of 21 *P. stutzeri* strains are publicly available (3–19). Comparative genome analysis and G+C content confirmed that strain ST-9 exhibited overall similarity to the *P. stutzeri* strains of genomovars previously sequenced. Average nucleotide identities based on BLAST (ANIb values) (20), which discriminated the genomovars of the species, confirmed the adscription of strain ST-9 to gv3: values higher than 96% were found for strains of the same genomovar, 80 to 93% for strains of different genomovars, and lower than 77% with other *Pseudomonas* species. The genome sequence will help to elucidate the chromosomal toluene degradation pathway of strain ST-9 and the mechanisms involved in the adaptation of *P. stutzeri* strains to contaminated sites.

### Nucleotide sequence accession numbers. 

This whole-genome shotgun project has been deposited at DDBJ/EMBL/GenBank under the accession no. JXJL00000000. The version described in this paper is version JXJL01000000.
